# More than Meets the Eye: Integration of Radiomics with Transcriptomics for Reconstructing the Tumor Microenvironment and Predicting Response to Therapy

**DOI:** 10.3390/cancers15061634

**Published:** 2023-03-07

**Authors:** Stella Logotheti, Alexandros G. Georgakilas

**Affiliations:** DNA Damage Laboratory, Physics Department, School of Applied Mathematical and Physical Sciences, National Technical University of Athens (NTUA), Zografou, 15780 Athens, Greece

For over a decade, large cancer-related datasets (big data) have continuously been produced and made publicly available to the scientific community. A current challenge is how the accumulating big data from several layers of biological organization, such as molecules, cells, tissues, and whole organs, can eventually be translated into optimized strategies for cancer management. These datasets are too large or complex to be dealt with by traditional data-processing application software and safely produce a meaningful and applicable conclusion. In this Editorial, we highlight the perspectives of combining medical imaging with transcriptional data and artificial intelligence methods for the prediction of response to therapy and the personalization of patient treatment.

Medical imaging represents a fundamental and well-established oncological examination. Radiologic modalities, such as computed tomography (CT), magnetic resonance imaging (MRI), positron emission tomography–computed tomography (PET/CT), or PET/MRI, are widely used in clinical practice to acquire spatial information on the lesion and surrounding tissues, assess tumor grade, and monitor a patient’s response to therapeutic regimens in a non-invasive manner. Intriguingly, beyond the typical medical image analysis and visual interpretation, the generated pictures contain details that are not perceived by the naked eye but can be highly informative for patient management. To extract a large number of undiscovered features from routinely acquired imaging data that cannot be captured by conventional means, a promising artificial intelligence-driven method, termed radiomics, has emerged at the interface of radiology and oncology. According to the concept of radiomics, images are more than pictures, they are data. Standing on the shoulders of computational science, radiomics approaches use algorithms that convert images to high-throughput, quantitative, and mineable data. The quantified spatial features of tumors are subsequently combined with the clinicopathological characteristics of patients and processed by sophisticated bioinformatics tools to develop models that aim to improve diagnostic, prognostic, and predictive accuracy. In this regard, the plethora of radiology images that are stored in the clinic archives represents a ‘goldmine’, whereby pictures can be revisited to generate clinical aids for the improvement of medical decisions [1].

The potential of radiomics for early tumor detection, prediction of patient survival, and/or assessment of response to various therapeutic modalities is collectively highlighted by an increasing number of studies. For example, Mahmood and colleagues recently combined radiomics with machine learning for detecting and classifying microcalcifications, i.e., indicators of potential carcinomas, in mammogram images. This approach showed superiority as compared to conventional diagnostic methods, thereby putting forth a tool that may aid radiologists for early breast cancer detection in regular clinical practice settings [2]. Moreover, a team led by Dr. Kontos at the University of Pennsylvania extracted and analyzed retrospectively the radiomic phenotypes from 110 CTs of lung adenocarcinoma patients and showed that integration with clinical data significantly improved the prediction of overall survival in stage III non-small-cell lung cancer (NSCLC) after chemoradiation [3]. Interestingly, the same group demonstrated that the readers’ level of training and clinical experience (e.g., data scientist, medical student, radiology trainee, or specialty-trained radiologist) does not influence the ability to extract accurate radiomic features for NSCLC on CT, suggesting that the method is user-friendly and can be reliably applied by a variety of health care professionals [4]. On a similar note, in a study published in *Cancers* on March 2022, the authors used prospectively collected longitudinal data from FDG-PET (positron emission tomography with 2-deoxy-2-[fluorine-18]fluoro-D-glucose), CT, and perfusion SPECT (single-photon emission computed tomography) images of NSCLC patients to investigate whether the multitask learning of multi-time point radiomic features can be used for improving survival outcome prediction. The method was compared to single-task learning and conventional clinical imaging feature model benchmarks. Multitask learning achieved higher survival prediction concordance compared with other modalities and models on pretreatment and mid-treatment FDG-PET images [5]. Intriguingly, an artificial intelligence-driven analysis of CT images of lesions from 203 patients with advanced melanoma and NSCLC undergoing checkpoint inhibition (anti-PD1) therapy showed that radiomic features may function as predictive biomarkers of response to immunotherapy with a potential to improve patient stratification [6]. In that sense, radiomic features were recently proposed as promising endpoints for clinical trials [7].

Nevertheless, we should keep in mind that tumors are heterogeneous structures, surrounded by a contexture of cellular (such as red blood cells, immune cells, fibroblasts, lipocytes) and acellular components (extracellular matrix, secreted signaling molecules), which are collectively described as the tumor microenvironment (TME). According to current notions, a neoplasm consists of highly versatile subpopulations of cells carrying genetic and epigenetic alterations, which arise constantly due to genomic instability. The cellular subpopulations express diversified transcriptional programs and eventually expand or contract in the neoplasm, in response to changes in their ΤΜΕ. Cell variants acquiring capabilities that offer selective advantages under specific microenvironmental changes have increased fitness and, thus, can adapt quickly to new conditions and evade therapeutic targeting [8]. Conversely, the TME can edit cancer cellular heterogeneity, and tumors that evolve under stronger immune pressure lose more immunogenic neoantigens, hence becoming less visible to the immune system [9]. In general, cancer progression is perceived as a highly dynamic and complex process, which follows Darwinian laws, and is shaped by combinations of phenotypic features acquired by the cancer cells and their interactions with the host microenvironment and immune system [8]. Tumor heterogeneity is a recognized intrinsic barrier to the efficacy of several cancer therapies, including next-generation immunotherapeutics, where acquired resistance is often manifested as antigen escape and immunosuppression [10]. To shed more light on the interplay of diverse tumor cell subpopulations with changing TME, high-throughput profiling platforms, such as transcriptomics, proteomics, and multispectral imaging flow cytometry, are increasingly being recruited. The advent of RNA sequencing (RNAseq) technology has enabled the comprehensive profiling of cellular heterogeneity, providing a basis for a deeper understanding of tumor–TME interplay. For example, single-cell RNAseq was recently used to map the cell type-specific transcriptome landscape of tumors and their microenvironment by analyzing tissue biopsies from advanced NSCLC patients [10]. Such studies can offer invaluable insights into cancer–TME crosstalk and its impact on therapeutic response.

In light of the aforementioned notions, it is reasonable to envisage that the molecular and cellular heterogeneity of tumors and their surrounding microenvironment may be, to an extent, imprinted in the radiological images in the form of patchiness not perceived by the human eye. Subtle visual inconsistencies in a 3D image of a lesion could possibly indicate sites of underlying molecular plasticity and/or tumor–TME interactions, which may fuel the tumor evolutionary trajectories toward disease progression and resistance to therapy. To this end, the integration of radiomic features with transcriptomic profiles theoretically provides a means to capture in situ the dynamic interplay of the tumor cell populations and the TME relative to space and time. The concept of ‘radiotranscriptomics’ was first introduced by Catrib and colleagues at the University of California Los Angeles (UCLA) to describe the synergy of imaging and transcriptomics in clinical assessment [11]. Unexpectedly, the COVID-19 pandemic crisis provided a fertile ground for international researchers to team up and develop, for the first time, a comprehensive artificial intelligence-driven radiotranscriptomics pipeline. In particular, in a prospective study led by Dr. Antoniades, ORFAN (Oxford Risk Factors and Non-Invasive Imaging Study) investigators in collaboration with the COMBAT consortium extracted radiomic features from routine CT angiograms and subsequently used machine learning to train the imaging data against arteries transcriptomic profiles, obtained via the RNA sequencing of respective biopsies. They subsequently developed a platform that not only predicts thrombosis and the likelihood of death in COVID-19 patients but also enables the identification of patients who are likely to respond well to dexamethasone [12]. This ‘pandemic heritage’ can be used as a springboard to design analogous radiotranscriptomics pipelines in the oncology setting.

Radiotranscriptomics have thus emerged as a potentially powerful new strategy for the development of non-invasive imaging biomarkers and the support of clinical decisions. The field is still in its infancy, offering both unprecedented opportunities and methodological challenges in the design of multidisciplinary workflows for the construction of accurate predictive models. The amount of meaningful information obtained depends on multiple factors, such as the ability of the medical imaging devices to generate high-resolution pictures, the performance of the transcriptomics platforms, and the robustness of the artificial intelligence methods. First of all, one consideration would be to discriminate image background noise from visual irregularities that may represent clinically relevant details. For example, the fast acquisition of MRI images may reduce the resolution of images, thereby jeopardizing the quality of the extracted radiomic features. Advantageously, new scanning devices include deep-learning (DL) solutions to save acquisition time. These built-in DL algorithms were recently shown to be able to reconstruct such low-resolution and noised MRI images into high-quality images, where radiomic features are restored [13]. Second, sophisticated algorithms are required for combining radiomic with molecular features to allow for the in situ identification of tumor–TME interactions on radiological images. Advantageously, a toolkit of computational methods that, in principle, combine spatial with molecular information to assign cell types with distinct RNA readouts to their locations in histological sections has already been developed in the context of spatial transcriptomics [14], providing a basis for extrapolation of these algorithms in the radiotranscriptomics field. It is of note that while a few studies have linked radiomic features with transcriptomics of bulk tumors [15,16], to date, no approach has specifically utilized transcriptomic data from individual cells/cell subpopulations, which would more accurately reflect the heterogeneity of the tumor and its microenvironment. Therefore, it is worthwhile to develop relevant workflows that include single-cell or single-nuclei RNAseq data of tumor biopsies. Software technology that is designed to simultaneously identify and track persons in a crowd [17] and/or tag individuals in large groups of animals [18] could perhaps be repurposed to trace several important cell types (e.g., activated T-cell populations) in a radiological image based on their specific molecular profiles. Such an approach would catalyze the detailed mapping of the interactions of tumors with TME that could be manipulated toward inducing durable responses to therapy. Future advancements in imaging technologies and RNAseq platforms, as well as in silico methods, may facilitate overlaying ‘-omics’ data from divergent levels of biological organization for the construction of cutting-edge models with high clinical and translational value for patient care.
